# Challenges of implementing multiple imputation to address missing data in economic evaluations

**DOI:** 10.1186/1745-6215-16-S2-P26

**Published:** 2015-11-16

**Authors:** Aida Moure Fernandez, Kirsty Garfield, Sian Noble, Daisy Gaunt, Laura Howe, Debbie Lawlor, William Hollingworth

**Affiliations:** 1 School of Social and Community Medicine, University of Bristol, Bristol, UK; 2 Bristol Randomised Trials Collaboration, Bristol, UK

## Background

Missing cost and outcomes data is a common problem for health economists analysing RCTs. Systematic reviews of the economic evaluation literature suggest that many analysts use crude methods (e.g. complete case analysis or single imputation) which are vulnerable to bias and may underestimate uncertainty. Multiple imputation (MI) is more appropriate if data are missing at random, but raises a number of methodological and practical challenges. Two case study RCTs assessing the cost-effectiveness of new interventions are used to explore these challenges.

## Methods

The first RCT is a small trial (n=100) of an intervention which aims to improve physical function in patients with a chronic condition. The second is a cluster RCT (n=2221 participants; n=30 clusters) of an intervention which aims to instil healthy practices related to nutrition and exercise in primary school children. We describe challenges at each stage of analysis.

## Results

The challenges discussed will include: understanding the prevalence and pattern of missing data; selecting appropriate imputation methods; identifying variables to include in the model and stratifying by random allocation; imputing non-normally distributed costs and bounded EQ-5D values; achieving convergence; accounting for clustering (i.e. multilevel MI); bootstrapping to obtain accelerated bias-corrected confidence intervals; and conducting sensitivity analyses.

## Conclusions

We use these case studies to address the challenges of implementing current guidelines for handling missing data in cost-effectiveness analysis as well as to identify gaps in current knowledge and priorities for future research.

